# Transrectal *vs*. transvaginal oocyte retrieval in patients requesting egg freezing for fertility preservation: a prospective observational study

**DOI:** 10.3389/fmed.2026.1761079

**Published:** 2026-07-03

**Authors:** Nagwan Ahmed Bahgat, Shamia Mohamedain Abdelgadir Younis, Rehaballa Mohamed Alkatary, Abdallah Mohammed Ahmed Ibrahim Mohammed, Elsamwael Elhakim, Mahmoud Thabet, Rayan G. Albarakati, Dalia Mahmoud Abdelmonem Elsherbini

**Affiliations:** 1 Department of Obstetrics and Gynecology, Faculty of Medicine, Mansoura University, Mansoura, Egypt; 2 Department of IVF and Embryology, Ferticlinic Fertilization Center, Abu Dhabi, United Arab Emirates; 3 Department of Obstetrics and Gynecology, Delta University for Science and Technology, Belkas, Egypt; 4 Al Sharq Obstetrics & Gynecology Department, Al Sharq Hospital, Fujairah, United Arab Emirates; 5 Department of Clinical Medical Sciences, College of Medicine, AlMaarefa University, Riyadh, Saudi Arabia; 6 Research Center, Deanship of Scientific Research and Post-Graduate Studies, AlMaarefa University, Riyadh, Saudi Arabia; 7 Department of Clinical Laboratory Sciences, College of Applied Medical Sciences, Jouf University, Sakaka, Saudi Arabia; 8 Department of Anatomy, Faculty of Medicine, Mansoura University, Mansoura, Egypt

**Keywords:** egg freezing, fertility preservation, intact hymen, transrectal oocyte retrieval, transvaginal oocyte retrieval

## Abstract

**Background and objectives:**

Oocyte freezing can be performed for up to ten years, with regulations regarding storage duration and age limits varying by country, including a maximum age of up to 50 years in some regions. The standard method for oocyte retrieval is transvaginal ultrasound; however, it is not suitable for patients with an intact hymen, particularly in regions with cultural and religious considerations, such as the Middle East. For these patients, transrectal ultrasound-assisted retrieval represents a safe alternative. This study evaluates transrectal oocyte retrieval and compares it with conventional ultrasound-guided transvaginal aspiration.

**Materials and methods:**

This prospective observational study involved 120 patients undergoing oocyte cryopreservation, divided into two groups: transrectal oocyte retrieval (TROR) for patients with intact hymens, and transvaginal oocyte retrieval (TVOR) for those without. The study compared follicle and oocyte retrieval counts between these groups, using regression analyses to evaluate the impact of variables such as age, body mass index (BMI), anti-Müllerian hormone (AMH), anesthesia duration, procedural time, and peak estradiol (E2) on the triggering day.

**Results:**

Number of oocytes correlated negatively with age in TROR and TVOR groups (*r* = −0.10, −0.55, *P* = 0.22, *P* < 0.001, respectively). It also correlated negatively with BMI in both groups (*r* = −0.20, −0.12, *P* = 0.03, *P* = 0.19, respectively). Number of oocytes showed a significant strong positive correlation with AMH level, anesthesia time, surgery time, peak E2 on triggering day, and number of follicles in the TROR group (*r* = 0.80, 0.56, 0.55, 0.81, 0.69, *P* < 0.001). Similarly, it showed significant moderate correlation with AMH (*r* = 0.40, *P* < 0.01) and strong correlation with anesthesia time, surgery time, peak E2 on triggering day, and number of follicles in the TVOR group (*r* = 0.73, 0.74, 0.80, 0.85, *P* < 0.001).

**Conclusion:**

Transvaginal oocyte collection is the standard and easiest method for oocyte retrieval. However, for patients with an intact hymen particularly in regions with cultural and religious considerations, trans-rectal oocyte collection is also regarded as a safe and simple alternative.

## 1 Introduction

Fertility decline or loss can be a result of natural or iatrogenic causes. Women can lose their ovarian reserve due to several factors, such as exposure to radiotherapy and chemotherapy as a treatment option for cancers, as the incidence of malignancy in women of reproductive age is about 10% (1). Exposure to gonadotoxic medications in patients diagnosed with autoimmune diseases may similarly impact ovarian reserve. Additionally, individuals undergoing surgical excision of ovarian tissue due to endometriosis or dermoid cysts may also face a decline in fertility (2). Chromosomal disorders, such as Turner syndrome, can lead to premature ovarian failure. Additionally, other genetic disorders that run in families may reduce ovarian reserve and shorten the reproductive period for these women. The effect of age on female fertility is widely recognized, especially in cases where women postpone motherhood due to career commitments, financial constraints, or the absence of a suitable partner (3). Due to these factors and the improved survival rates following cancer treatments, oocyte cryopreservation has become a significant assisted reproductive technology (ART) that doctors recommend before initiating chemotherapy, radiotherapy, cytotoxic medication, and surgery involving ovarian tissues. Patients also require it to preserve their fertility and delay maternity for non-medical reasons. In 2013, the American Society for Reproductive Medicine (ASRM) approved its use for fertility preservation in oncology patients. But in 2018, the ASRM practice committee accepted non-medical egg freezing as ethically permissible and termed it as “planned oocyte cryopreservation” ("planned OC”) (4–6).

Starting the cycles for egg freezing should be initiated as soon as possible for cancer patients. In contrast, for patients undergoing elective surgeries or those with autoimmune diseases, the process can wait until the next menstrual cycle. For planned oocyte cryopreservation, the timing of the cycles should be arranged based on patient requests and in coordination with their healthcare providers (7). The ovarian stimulation protocol for egg freezing cycles depends on the anti-mullerian Hormone (AMH) level, patient age and weight, and reason for oocyte cryopreservation (8). Vitrification, or rapid freezing, is the best way to freeze oocytes because it lowers the chance of oocyte damage from the ice crystals that form in the slow freezing method (9). The number of oocytes required for optimum results was suggested according to each age category, with 20 mature oocytes being the best (3). Oocyte freezing is allowed for up to ten years, but each country has its own rules about how long they can be frozen and how old a person must be to use them. In some countries, it is allowed until the age of 50 (10). The method of oocyte collection is a crucial factor in the success of the oocyte freezing cycle. It is widely recognized that the transvaginal approach is the simplest and most commonly employed technique. However, in specific circumstances, such as significantly displaced ovaries due to large fibroids or severe adhesions following pelvic surgery or cesarean sections, *in vitro* fertilization (IVF) practitioners may opt for transabdominal oocyte retrieval. This method is considerably more challenging and entails greater potential risks compared to the transvaginal approach. It also needs a more experienced doctor, a better-prepared operating room, and laparoscopic surgeons on hand in case something goes wrong and needs to be fixed right away (11). Laparoscopic oocyte aspiration is an invasive procedure compared to the transvaginal method. It poses greater risks of vascular and internal organ trauma, increased bleeding, a challenging approach, internal visualization of the follicles, the necessity for a proficient laparoscopic surgeon, extended surgery duration, anesthesia complications, and an extended hospital stay (12). The presence of an intact hymen may interfere with the required transvaginal ultrasound-guided oocyte aspiration and hence the acceptability of oocyte collection for freezing, especially in some cultures and areas, like, for example, the Middle East. Urosurgeons have long practiced transrectal prostatic biopsy with minimal or no reported complications (13). Why not perform oocyte retrieval in a way that avoids hymen disruption, allowing patients in these areas the opportunity to freeze their oocytes for fertility preservation? We began performing ultrasound-guided transrectal oocyte collection to prevent disrupting the hymen for patients who wish to keep it intact. To the authors' knowledge, there is a paucity of research on TROR, with just one study conducted in animals (14), one research in humans (15), and one case series (16). The population considered in our study included all women with intact hymen in the Middle East and Far East who can't do transvaginal ultrasounds to keep intact hymen due to cultural and religious concerns, and they are millions. This study supports fertility preservation in women without breaching their cultural and religious concepts. This is the first study comparing the efficacy of the TROR vs. TVOR oocyte retrieval methods. In this study, we aim to evaluate the transrectal oocyte collection and compare it to a traditional ultrasound-guided transvaginal aspiration.

## 2 Subjects and methods

### 2.1 Study design/setting and population

This prospective observational (non-randomized) study was conducted in a large *in vitro* fertilization (IVF) center in Abu Dhabi, United Arab Emirates, between February 2024 and February 2025. Ethical approval was obtained from the Research Ethics Committee of the FertiClinic Fertilization Center (REC-APP-2024-001), and all participants provided informed consent prior to inclusion. The study comprised 120 patients who sought egg freezing for the purpose of fertility preservation.

The patients were split into two groups based on clinical indication and patient preference, rather than randomization. The first group, the transrectal oocyte retrieval group (TROR), had patients with intact hymens who wanted to freeze their eggs without hymen disruption. The second group is made up of patients who don't have an intact hymen and want to freeze their eggs the traditional way, through the transvaginal way. This group is called the transvaginal oocyte retrieval group (TVOR). All participants underwent a comprehensive pelvic assessment prior to the procedure with transabdominal ultrasound to evaluate ovarian accessibility, follicular distribution, and spatial relationships between the ovaries and surrounding structures. The feasibility for transrectal oocyte retrieval (TROR) was evaluated based on the ability to visualize a safe needle path from the rectal wall to the ovary without the interposition of critical structures. In situations where transabdominal ultrasound was insufficient to confidently assess feasibility, especially in participants with elevated body mass index (BMI), transrectal ultrasound was performed pre-procedurally to better delineate pelvic anatomy and confirm safe access.

The following criteria were applied for offering oocyte cryopreservation in accordance with European Society of Human Reproduction and Embryology **(**ESHRE) guidance on female fertility preservation (17). Patients were enrolled based on presentation and eligibility criteria, without selection based on ovarian reserve parameters. The inclusion criteria included post-menarche status, medical fitness for the procedure, and women requesting oocyte cryopreservation for age-related fertility loss without history of autoimmune diseases, endometriosis, ovarian tumors, cancer, or smoking. Exclusion criteria included premenarche status; being medically unfit for the procedure (such as large uterine fibroids, ovarian cysts, aplastic anemia, and significant gastrointestinal comorbidities like active ulcerative colitis); and insufficient time to complete oocyte cryopreservation.

All our patients had a first visit where they were counseled and informed about the stimulation protocols, medications, side effects, follow-up, what to expect, the egg collection procedures, anesthesia, the outcome of the procedure, and follow-up after the procedure. All the patients who were included wanted to freeze their eggs for non-medical reasons, so they chose when to start.

There was no formal matching between groups as in real-life clinical practice; baseline demographic and clinical characteristics were recorded for all participants, and relevant variables including anti-mullerian hormone (AMH) were used in multivariable regression analysis to adjust for possible confounding effects of baseline differences between groups.

### 2.2 Ovarian stimulation

The standard protocol for ovarian stimulation in both groups involved a short gonadotropin-releasing hormone (GnRH) antagonist cycle, as previously described (18), which utilized recombinant subcutaneous FSH (Gonal-F; Merck Serono, Spain), recombinant FSH with LH (Pergoveris; Merck Serono), or highly purified human menopausal gonadotropin (Merional; Ferring, Germany) and was initiated on day 2 of the menstrual cycle. However, some patients with endometriosis started on the long protocol.

The initial dose of gonadotropin was individualized for each patient according to age, BMI, antral follicle count, anti-mullerian hormone (AMH) concentration, and previous responsiveness to ovarian stimulation. After one week, follow-up for follicle size was through transabdominal ultrasound and hormone level in the first group, but in some cases with high BMI, we did a transrectal scan after taking permission and explaining to the patient. Established clinical practice endorses the utilization of a transrectal scan, which has shown equivalent diagnostic efficacy for ovarian evaluation in similar cohorts. The transrectal probe is closer to the pelvic organs, which makes the picture sharper. Transabdominal scanning makes the picture less clear when there is more adipose tissue in subjects with high BMI (19). Patients in the second group were followed up with the standard transvaginal ultrasound after one week.

When the leading follicle reached 17 mm, the ovulation trigger was done with either recombinant human chorionic gonadotropin alfa 500 micrograms (Ovitrelle: Merck Serono) or gonadotropin-releasing hormone agonist 0.2 (GnRH-a, triptoreline acetate, Ferring) according to the number of follicles and estrogen levels. Patients are then scheduled for oocyte collection after 34–36 h (15).

### 2.3 Subjects preparation for the procedures

The first group of patients (TROR) had special preparation, which included cleaning the colon the day before the procedure to avoid any possible contamination and infection. One day before the procedure, the patient had to start taking a double antibiotic (amoxicillin-clavulanic acid 1-gram capsule and metronidazole 500 mg) and keep it for a week. They also had to stop eating solid food and only drink fluids. To empty the rectum, they had to use a colonic cleaning solution. Put the macrogol sachet in a cup of water and drink it over the course of 4 h. Do a rectal enema at least 4 h before the procedure. The patient was given written instructions to ensure they understood and followed them. Bowel preparation was performed according to institutional clinical practice based on principles of other transrectal procedures such as prostate biopsy and colonoscopy (20, 21). However, this approach has not been specifically validated for transrectal oocyte retrieval, and its necessity and sufficiency remain to be determined.

### 2.4 Day of the procedures

Patients in both groups were instructed to fast before the procedure for 8 h. On the day of the procedure, patients came fasting, in the operative theater, and under sterile conditions; conscious sedation was given, and they were placed in the lithotomy position. For the first group (TROR), rectal cleaning was done using betadine solution with small gauze on a small sponge holder, then cleaning with saline several times till full rectal cleaning was achieved. Then, using a transrectal probe that is covered with a sterile cover and needle holder or using the transvaginal probe with a needle holder, slowly advance it in the rectum till the ovaries clearly appear with follicles near to the probe, and be sure there is no structure in between, for example, the cervix. We start oocyte collection with a single- or double-lumen needle with suction pressure of about 100 mmHg. In case there are fewer than 4 follicles, we do oocyte flushing and curettage in order to get the maximum number of oocytes available for egg freezing. Follicle fluid is aspirated to be handed to the embryologist for oocyte checking and evaluation to start oocyte freezing. After all follicle aspiration probes are retracted from the rectum slowly after pelvic evaluation to exclude any bleeding internally, observe the rectum and anus for any bleeding externally (15). On discharge, all women were advised to continue the antibiotics started before the procedure for another five days. A follow-up visit after 5 days is mandatory to establish whether the patients encountered any complications post-procedure.

The second group (TVOR) will undergo standard transvaginal oocyte collection. This procedure involves sedation, cleaning the vaginal area with saline, and using a transvaginal probe that is covered with a sterile cover and has a needle holder attached. The probe will be advanced into the vaginal fornixes to initiate oocyte collection, utilizing either a single- or double-lumen needle in the standard manner. The embryologist will receive the follicular fluid to inspect for oocytes, begin denudation, and proceed with oocyte freezing. After retrieval of the oocytes, patients were monitored in the recovery area according to standard clinical practice until the established criteria for discharge were met (hemodynamic stability, adequate recovery from anesthesia, and absence of immediate complications). The length of recovery was individualized according to the patient's condition and ranged usually from 1 to 6 h (22). Postoperative intra-abdominal bleeding was systematically assessed using immediate post-procedural ultrasound to detect the presence of free fluid within the pelvis. In addition, participants were clinically monitored for signs and symptoms suggestive of bleeding, including abdominal pain, decreased blood pressure, and tachycardia.

### 2.5 Outcome measures

The study was conducted to evaluate the transrectal procedure in comparison to the standard transvaginal oocyte collection. The main points of comparison include the number of follicles and the number of oocytes retrieved in both groups. A regression study for predictor variables, including age, BMI, AMH, duration of anesthesia, surgery time, and peak estradiol (E_2_), on the triggering day and their impact on the number of follicles and oocytes was conducted. The accessibility of the ovaries and the number of possible complications—whether intraoperative, such as bleeding or organ trauma, or postoperative, including pain, fever, infection, bleeding, injury, and changes in bowel habits—were compared in both groups.

### 2.6 Statistical analysis and data interpretation

Data analysis was performed by SPSS software, version 26 (SPSS Inc., PASW Statistics for Windows version 26). Chicago: SPSS Inc. Qualitative data were described using numbers and percentages. Quantitative data were described using median and interquartile range (IQR) for non-normally distributed data after testing normality using the Kolmogorov-Smirnov test. The significance of the obtained results was judged at the (0.05) level. The Fisher exact test was used to compare qualitative data between groups as appropriate. The Mann-Whitney U test was used to compare the two studied groups for non-normally distributed data. The Mann-Whitney effect size (r) was calculated using the formula r = Z/sqrt(*n*), where n represents the number of cases. Pearson's correlation analysis of the key outcome variables was conducted. To study the association of outcome predictors with the number of follicles and oocytes, simple linear regression analysis was conducted to calculate the crude regression coefficient with a 95% confidence level and *P*-values, testing the association between dependent and independent variables. Selection of variables for the multiple linear regression model was based on a combination of clinical relevance and statistical criteria. Variables demonstrating a *P* < 0.25 in simple linear regression were considered for inclusion in the multivariable model to avoid missing important variables that may not reach statistical significance after adjustment for confounding variables. Adjusted regression coefficients with 95% confidence intervals and *P*-values were reported. Multicollinearity among independent variables was assessed prior to model fitting using variance inflation factors (VIF). Factors with VIF > 5 were excluded from the analysis. Statistical significance in the final model was set at *P* < 0.05. GraphPad Prism version 9 was used to plot the graphs.

## 3 Results

**Figure 1:**
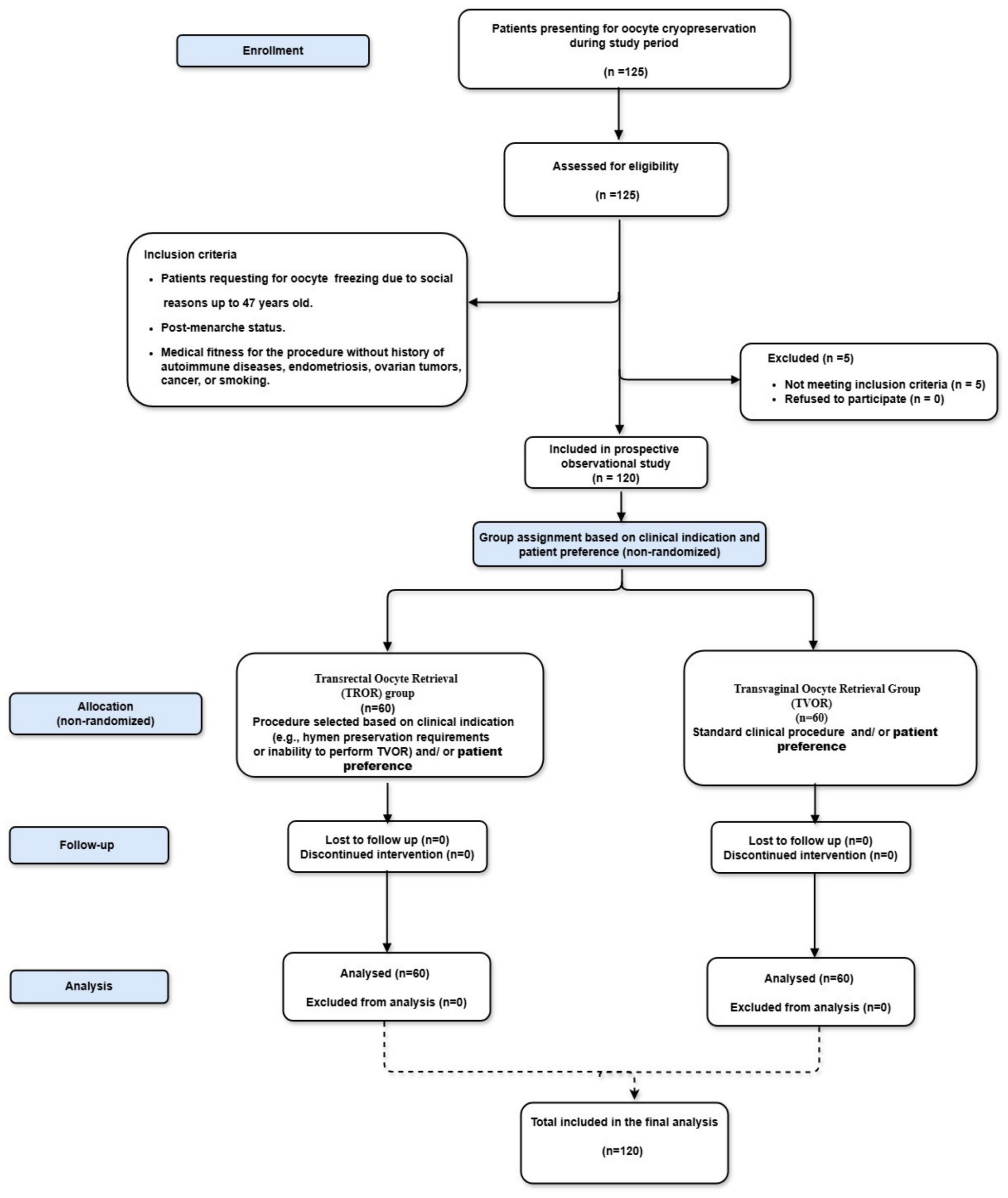
Flow diagram of the study population.

The study included 120 patients who requested egg freezing for fertility preservation. The patients were divided into two groups. The first group, the transrectal oocyte retrieval group (TROR), included patients with intact hymens requesting egg freezing without hymen disruption. The second group, the transvaginal oocyte retrieval group (TVOR), are patients without an intact hymen requesting egg freezing through the traditional transvaginal way (Figure 1).

Table 1 showed no significant differences in age or BMI between the two groups, with no or small effect sizes (*r* = 0.045 and 0.176, respectively). The median AMH level in the TVOR group was significantly higher (*P* < 0.01), with an intermediate effect size (*r* = 0.288), compared with the TROR group. The median anesthesia time in the TVOR group was also significantly higher (*P* = 0.04), with a small effect size (*r* = 0.184), compared with the TROR group. There was no significant difference in surgery time between the two groups (*P* = 0.06), with a small effect size (*r* = 0.179).

**Table 1:** Patient criteria.

Variables	Transrectal oocyte retrieval group *N* = 60	Transvaginal oocyte retrieval group *N* = 60	Effect size (*r*)	*P* value
	Median (IQR)			
Age(years)	38.70 (37.00–41.75)	40.00 (34.00–43.00)	0.045	0.64 (ns)
BMI (kg/m^2^)	26.35 (24.63–28.40)	27.70 (25.60–30.18)	0.176	0.05 (ns)
AMH (ng/ml)	0.42 (0.082–1.075)	0.77 (0.177–3.200)	0.288	< 0.01^**^
Anesthesia time/min	6.00 (5.00–7.00)	7.00 (5.25–8.00)	0.184	0.04^*^
Surgery time/min	3.00 (2.00–4.00)	4.00 (2.25–5.00)	0.179	0.06 (ns)

For numerical data, median and interquartile range (IQR) using the Mann-Whitney *U*-test was applied, *P* < 0.05^*^ and 0.01^**^ is statistically significant. BMI, body mass index; AMH, anti-mullerian hormone.

Table 2 shows the median stimulation length in the TROR and TVOR groups as 10 and 11 days, respectively (*P* = 0.60, *r* = 0.045). The median peak E2 levels on the triggering day were 878.0 and 932.0 pg/mL, respectively (*P* = 0.59, r = 0.045). The median number of follicles was 4 and 5, respectively (*P* = 0.08 and *r* = 0.158). The median number of oocytes retrieved was 4 in both groups (*P* = 0.47, *r* = 0.063). Similarly, the median number of mature oocytes II was 4 in both groups (*P* = 0.49, *r* = 0.063). Regarding the stimulation protocol, there was no significant difference between the two groups (Fisher's exact test = 2.03, *P* = 0.50).

**Table 2:** Comparison of stimulation protocol, laboratory and IVF between studied groups.

Variables	Transrectal oocyte retrieval group *N* = 60	Transvaginal oocyte retrieval group *N* = 60	Effect size (*r*)	*P* value
	Median (IQR)			
Stimulation duration (days)	10 (9–13)	11 (9–13)	0.045	0.60 (ns)
Peak E2 (pg/mL)	878.0 (269.5–1,456)	932.0 (320.0–2,419)	0.045	0.59 (ns)
Number of follicles	4 (2–6)	5 (2–9)	0.158	0.08 (ns)
Number of oocytes retrieved	4 (2–8)	4 (2–12)	0.063	0.47 (ns)
Number of mature oocyte II (MII)	4 (2–6)	4 (1–10)	0.063	0.49 (ns)
	*N* (%)		**Fisher's exact tests**	***P*** **value**
Stimulation protocol			2.03	0.50 (ns)
Long	2 (3.3)	0 (0)		
Antagonist	58 (96.7)	60 (100)		

For numerical data, median and interquartile range (IQR) using the Mann-Whitney *U*-test was applied, *P* > 0.05 (ns) is statistically non-significant. E2: estradiol on triggering day. The qualitative nominal data were expressed as frequency (%), and Fisher's exact test was applied; *P* > 0.05 (ns) is statistically non-significant.

**Figure 2:**
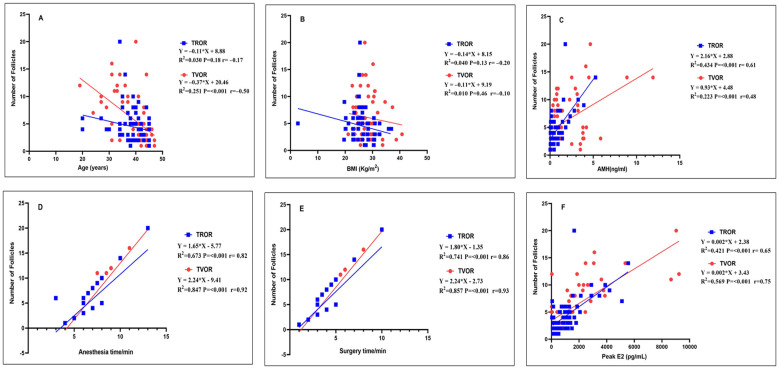
Factors affecting number of retrieved follicles by linear regression analysis: **(A)** Age, **(B)** BMI, **(C)** AMH, **(D)** Anesthesia time, **(E)** Surgery time, and **(F)** Peak E2.

Table 3 and Figure 2 present the results of correlational analyses between the number of retrieved follicles and several key outcome variables. The number of follicles was negatively correlated with age in both the TROR and TVOR groups (*r* = −0.17, *P* = 0.09; *r* = −0.50, *P* < 0.001, respectively). It was also negatively correlated with BMI in both groups (*r* = −0.20, *P* = 0.06; *r* = −0.10, *P* = 0.23, respectively). In the TROR group, the number of follicles showed significant strong positive correlations with AMH level (*r* = 0.61), anesthesia time (*r* = 0.82), surgery time (*r* = 0.86), and peak E2 on the triggering day (*r* = 0.65), all with *P* < 0.001. Similarly, in the TVOR group, the number of follicles showed a significant moderate correlation with AMH (r = 0.48, *P* < 0.001) and strong correlations with anesthesia time (*r* = 0.92), surgery time (*r* = 0.93), and peak E2 on the triggering day (*r* = 0.75), all with *P* < 0.001.

**Table 3:** Factors affecting number of retrieved follicles by linear regression analysis.

Variable(s)	Correlation parameters		Simple linear regression		Multiple linear regression	
	Correlation coefficient (*r*)	*P*-value	B^a^ (95% CI)	*P*-value	B^b^ (95% CI)	*P*-value
Transrectal oocyte retrieval group						
Age (years)	−0.17	0.09	−0.11 (−0.28, 0.06)	0.18	−0.02 (−0.11, 0.07)	0.64
BMI (kg/m^2^)	−0.20	0.06	−0.14 (−0.32, 0.04)	0.13	−0.04 (−0.13, 0.06)	0.47
AMH (ng/ml)	0.61	**< 0.001** ^***^	2.16 (1.51, 2.81)	**< 0.001** ^***^	0.52 (−0.26, 1.30)	0.18
Anesthesia time/min	0.82	**< 0.001** ^***^	1.65 (1.35, 1.96)	**< 0.001** ^***^	1.27 (0.95, 1.59)	**< 0.001** ^***^
Surgery time/min	0.86	**< 0.001** ^***^	1.80 (1.51, 2.07)	**< 0.001** ^***^	–	–
Peak E2 (pg/mL)	0.65	**< 0.001** ^***^	0.002 (0.001, 0.002)	**< 0.001** ^***^	0.001 (0.00, 0.001)	0.11
Transvaginal oocyte retrieval group						
Age (years)	−0.50	**< 0.001** ^***^	−0.37 (−0.54, −0.20)	**< 0.001** ^***^	−0.09 (−0.16, −0.01)	**0.019** ^*^
BMI (kg/m^2^)	−0.10	0.23	−0.11 (−0.40, 0.19)	0.46	–	–
AMH (ng/ml)	0.48	**< 0.001** ^***^	0.93 (0.50, 1.41)	**< 0.001** ^***^	0.05 (−0.14, 0.25)	0.58
Anesthesia time/min	0.92	**< 0.001** ^***^	2.24 (1.99, 2.49)	**< 0.001** ^***^	1.68 (1.38, 1.99)	**< 0.001** ^***^
Surgery time/min	0.93	**< 0.001** ^***^	2.24 (2.00, 2.48)	**< 0.001** ^***^	–	–
Peak E2 (pg/mL)	0.75	**< 0.001** ^***^	0.002 (0.001, 0.002)	**< 0.001** ^***^	0.001 (0.000, −0.001)	**< 0.001** ^***^

B^a^ Crude regression coefficient, B^b^ Adjusted regression coefficient. Variables with *P*-value < 0.25 were adjusted for multiple regression analysis. *P* < 0.05^*^, 0.001^***^ is statistically significant. BMI, body mass index; AMH, anti-mullerian hormone; E2, estradiol.

Using simple linear regression, a decrease in age accounts for a 3% increase in the number of follicles in the TROR group and a 25.1% increase in the TVOR group (crude B = −0.11 and −0.37; *P* = 0.18 and *P* < 0.001; R^2^ = 0.030 and 0.251, respectively). Additionally, a decrease in BMI accounts for a 4% increase in the TROR group and a 1% increase in the TVOR group (crude B = −0.14 and −0.11; *P* = 0.13 and *P* = 0.46; R^2^ = 0.040 and 0.010, respectively). On the other hand, an increase in AMH level explained 43.4% and 22.3% of the variation in the number of follicles in the TROR and TVOR groups, respectively (crude B = 2.16 and 0.93; *P* < 0.001 for both; R^2^ = 0.434 and 0.223, respectively). An increase in anesthesia time explains the 67.3% and 84.7% increase in the number of follicles in the TROR and TVOR groups, respectively (crude B = 1.65 and 2.24; *P* < 0.001 for both; R^2^ = 0.673 and 0.847, respectively). An increase in surgery time explains the 74.1% and 85.7% increase in the number of follicles in the TROR and TVOR groups, respectively (crude B = 1.80 and 2.24; *P* < 0.001 for both; R^2^ = 0.741 and 0.857, respectively). An increase in peak E2 on the triggering day explains the 42.1% and 56.9% increase in the number of follicles in the TROR and TVOR groups, respectively (crude B = 0.002 for both; *P* < 0.001 for both; R^2^ = 0.421 and 0.569, respectively).

Multiple regression analysis revealed that only anesthesia time remained a significant independent predictor of the number of follicles in the TROR group (B = 1.27, 95% CI = 0.95–1.59, *P* < 0.001), while the other variables (age, BMI, AMH level, and peak E2) were not significantly associated after adjustment for confounders. It also revealed that 76.1% of the variation in the number of follicles in the TROR group is explained by age, BMI, AMH level, anesthesia time, and peak E2 on the triggering day, according to the multiple linear regression model (R^2^ = 0.761).

On the other hand, in the TVOR group, the multiple regression analysis revealed that age was significantly associated with the number of follicles, showing a negative relationship (B = −0.09, 95% CI = −0.16 to −0.01, *P* = 0.019). Anesthesia time demonstrated a strong positive association (B = 1.68, 95% CI = 1.38–1.99, *P* < 0.001). Peak E2 levels were also statistically significant (B = 0.001, *P* < 0.001). Anti-Müllerian hormone levels were not significantly associated with the outcome after adjustment (*P* = 0.58). It also revealed that 89.9% of variation in the number of follicles in TVOR groups is explained by age, AMH level, anesthesia time, and peak E2 on triggering day, according to the multiple linear regression model (R^2^ = 0.899).

**Figure 3:**
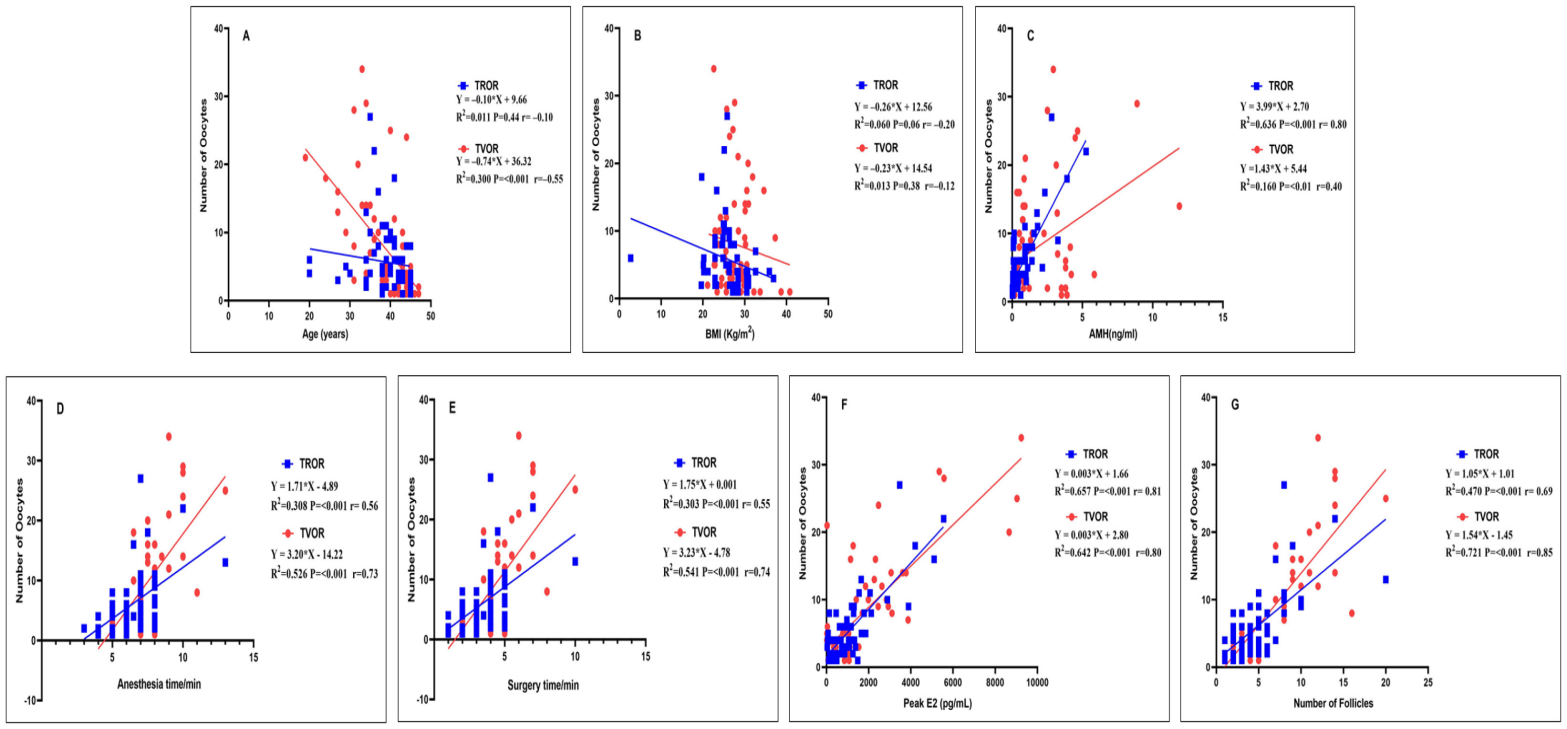
Factors affecting number of retrieved oocytes by linear regression analysis: **(A)** Age, **(B)** BMI, **(C)** AMH, **(D)** Anesthesia time, **(E)** Surgery time, **(F)** Peak E2, and **(G)** Number of follicles.

Table 4 and Figure 3 present the results of correlational analyses between the number of retrieved oocytes and several key outcome variables. Number of oocytes correlated negatively with age in TROR and TVOR groups (*r* = −0.10, −0.55, *P* = 0.22, *P* < 0.001, respectively). It also correlated negatively with BMI in both groups (*r* = −0.20, −0.12, *P* = 0.03, *P* = 0.19, respectively). Number of oocytes showed a significant strong positive correlation with AMH level, anesthesia time, surgery time, peak E2 on triggering day, and number of follicles in the TROR group (*r* = 0.80, 0.56, 0.55, 0.81, 0.69, *P* < 0.001). Similarly, it showed significant moderate correlation with AMH (*r* = 0.40, *P* < 0.01) and strong correlation with anesthesia time, surgery time, peak E2 on triggering day, and number of follicles in the TVOR group (*r* = 0.73, 0.74, 0.80, 0.85, *P* < 0.001).

**Table 4:** Factors affecting number of retrieved oocytes by linear regression analysis.

Variable(s)	Correlation parameters		Simple linear regression		Multiple linear regression	
	Correlation coefficient (*r*)	*P*-value	B^a^ (95% CI)	*P*-value	B^b^ (95% CI)	*P*-value
Transrectal oocyte retrieval group						
Age (years)	−0.10	0.22	−0.10 (−0.36, 0.16)	0.44	–	–
BMI (kg/m^2^)	−0.20	**0.03** ^*^	−0.26 (−0.53, 0.01)	0.06	−0.05 (−0.20, 0.10)	0.52
AMH	0.80	**< 0.001** ^***^	3.99 (3.20, 4.78)	**< 0.001** ^***^	1.64 (0.37, 2.90)	**0.012** ^*^
Anesthesia time/min	0.56	**< 0.001** ^***^	1.71 (1.03, 2.38)	**< 0.001** ^***^	0.13 (−63, 0.89)	0.73
Surgery time/min	0.55	**< 0.001** ^***^	1.75 (1.05, 2.45)	**< 0.001** ^***^	–	–
Peak E2 (pg/mL)	0.81	**< 0.001** ^***^	0.003 (0.003, 0.004)	**< 0.001** ^***^	0.002 (0.001, 0.003)	**< 0.01** ^**^
Number of follicles	0.69	**< 0.001** ^***^	1.05 (0.75, 1.34)	**< 0.001** ^***^	0.25 (−0.19, 0.68)	0.26
Transvaginal oocyte retrieval group						
Age (years)	−0.55	**< 0.001** ^***^	−0.74 (−1.04, −0.44)	**< 0.001** ^***^	−0.25 (−0.44, −0.07)	**< 0.01** ^**^
BMI (kg/m^2^)	−0.12	0.192	−0.23 (−77, 0.30)	0.383	–	–
AMH	0.40	**< 0.01** ^**^	1.43 (0.57, 2.30)	**< 0.01** ^**^	0.02 (−0.47, 0.50)	0.95
Anesthesia time/min	0.73	**< 0.001** ^***^	3.20 (2.40, 4.00)	**< 0.001** ^***^	–	–
Surgery time/min	0.74	**< 0.001** ^***^	3.23 (2.44, 4.01)	**< 0.001** ^***^	–	–
Peak E2 (pg/mL)	0.80	**< 0.001** ^***^	0.003 (0.002, 0.004)	**< 0.001** ^***^	0.001 (0.001, 0.002)	**< 0.001** ^***^
Number of follicles	0.85	**< 0.001** ^***^	1.54 (1.29, 1.80)	**< 0.001** ^***^	0.83 (0.45, 1.21)	**< 0.001** ^***^

B^a^ Crude regression coefficient, B^b^ Adjusted regression coefficient. Variables with *P*-value < 0.25 were adjusted for multiple regression analysis. *P* < 0.05^*^, 0.01^**^, and 0.001^***^ is statistically significant. BMI, body mass index; AMH, anti-Mullerian hormone; E2, estradiol.

By simple linear regression, a decrease in age explains a 1.1% and 30% increase in number of follicles in TROR and TVOR groups, respectively (crude B = −0.10, −0.74, *P* = 0.44, *P* < 0.001, R^2^ = 0.011, 0.300, respectively), and a decrease in BMI explains a 6% and 1.3% increase in the number of follicles in TROR and TVOR groups (crude B = −0.26, −0.23*, P* = 0.06, *P* = 0.38, R^2^ = 0.060, 0.013, respectively). On the other hand, an increase in AMH level explains a 63.3% and 16% increase in the number of oocytes in the TROR and TVOR groups, respectively (crude B = 3.99 and 1.43, *P* < 0.001 and *P* < 0.01, and R^2^ = 0.636 and 0.160, respectively). An increase in anesthesia time explains the 30.8% and 52.6% increases in the number of oocytes in the TROR and TVOR groups, respectively (crude B = 1.71 and 3.20, *P* < 0.001 and *P* < 0.001, R^2^ = 0.308 and 0.526, respectively). An increase in surgery time explains the 30.3% and 54.1% increase in the number of oocytes in the TROR and TVOR groups, respectively (crude B = 1.75 and 3.23, *P* < 0.001 and *P* < 0.001, and R^2^ = 0.303 and 0.541, respectively). An increase in peak E2 on the triggering day explains the 65.7% and 64.2% increase in the number of oocytes in the TROR and TVOR groups, respectively (crude B = 0.003, 0.003; *P* < 0.001, *P* < 0.001; R^2^ = 0.657, 0.642, respectively). An increase in the number of follicles explains the 47% and 72.1% increase in the number of oocytes in the TROR and TVOR groups, respectively (crude B = 1.05 and 1.54, *P* < 0.001 and *P* < 0.001, R^2^ = 0.470 and 0.721, respectively).

In the TROR group, multiple regression analysis demonstrated that AMH and peak E2 levels were significant independent predictors of the number of oocytes retrieved. AMH showed a positive association with oocyte yield (B = 1.64, 95% CI: 0.37–2.90, *P* = 0.012). Similarly, peak E2 levels were significantly associated with oocyte yield (B = 0.002, 95% CI: 0.001–0.003, *P* < 0.01). In contrast, BMI was not significantly associated with the number of oocytes retrieved (B = −0.05, 95% CI: −0.20 to 0.10, *P* = 0.52). Anesthesia time also did not demonstrate a significant effect on oocyte yield (B = 0.13, *P* = 0.73). Furthermore, the total number of follicles was not an independent predictor after adjustment (B = 0.25, 95% CI: −0.19 to 0.68, *P* = 0.26). It also revealed that 73.7% of the variation in the number of oocytes in the TROR group is explained by BMI, AMH level, anesthesia time, E2 on the triggering day, and number of follicles (R^2^ = 0.737).

On the other hand, in TVOR group, the multiple regression analysis revealed that age, peak E2 levels, and total number of follicles were significant predictors of oocyte yield, whereas AMH was not independently associated after adjustment. Age showed a significant negative association with number of oocyte (B = −0.25, 95% CI: −0.44 to −0.07, *P* < 0.01), peak E2 levels were significantly and positively associated with the number of oocyte (B = 0.001, 95% CI: 0.001–0.002, *P* < 0.001). The total number of follicles was also significantly associated with the number of oocyte (B = 0.83, 95% CI: 0.45–1.21, *P* < 0.001). In contrast, AMH was not significantly associated with the number of oocyte (B = 0.02, 95% CI: −0.47 to 0.50, *P* = 0.95), suggesting no independent predictive value after adjustment. It also revealed that 80.7% of variation in the number of oocytes in TVOR groups is explained by age, AMH level, anesthesia time, peak E2 on triggering day, and number of follicles (R^2^ = 0.807).

Regarding access to the ovaries in both groups, we did not face any problems in either group. Regarding intraoperative and postoperative complications, we did not report any in both groups (Table 5).

**Table 5:** Intraoperative and postoperative complications.

	Transrectal oocyte retrieval group	Transvaginal oocyte retrieval group
Intraoperative complications		
Trauma	0	0
Bleeding	0	0
Cancellation	0	0
Anesthesia complication	0	0
Postoperative complications		
Bleeding	0	0
Fever	0	0
Pain	0	0
Infection	0	0
Hospital admission	0	0

## 4 Discussion

Oocyte freezing is recommended for fertility preservation due to different reasons, whether medical or non-medical (23). Different approaches can facilitate the oocyte pick-up, which is the most critical step. Transvaginal ultrasound-assisted oocyte retrieval is the standard method, as it is proven to be the safest, easiest, and least complicated (24). However, this technique may not be feasible in certain patient populations. This includes patients with an intact hymen, particularly in regions where cultural and religious considerations preclude transvaginal access, such as the Middle East. For such patients, transrectal ultrasound-assisted oocyte retrieval is the safe and accepted way (15).

Beyond this setting, TROR may also have wider application in patients with anatomic or surgical barriers to transvaginal access. This includes transgender men (female-to-male), especially those who have undergone or are planning gender-affirming procedures such as vaginectomy, where transvaginal access is not possible. In these settings, TROR may be a clinically feasible, minimally invasive alternative to laparoscopic oocyte retrieval for fertility preservation. As transgender healthcare and oncofertility continue to evolve, such approaches may play an increasingly important role in expanding access to fertility preservation options equitably, in line with current recommendations from international societies supporting fertility preservation in transgender individuals [e.g., ESHRE, ASRM and The World Professional Association for Transgender Health (WPATH)] (17, 25, 26).

Another method of oocyte retrieval is transabdominal follicular aspiration, which could be considered in patients requiring an intact hymen or in cases when ovarian access is limited due to anatomical factors (27). However, it is a more sophisticated procedure due to limited follicular view, high body mass index, presence of organs, difficulty seeing the end of the needle, and possibility of organ or vessel injury. Laparoscopic oocyte retrieval was also suggested (12), but the view of the stimulated ovary reveals a challenge in getting into the follicles and aspirating the oocyte, which is less commonly used due to technical limitations. In addition, the need for a highly specialized laparoscopic surgeon, a well-prepared operating theater, and general anesthesia is considered riskier for the patient.

In our study we compared the standard transvaginal route with the new transrectal route. We found that no significant difference was found between the two groups, as TROR showed comparable reproductive outcomes to TVOR. We evaluated the complications that might occur from the ovum pickup. No intraoperative or postoperative complications were observed in either group, supporting the safety of TROR as transvaginal oocyte retrieval. These findings are in correlation with findings by a study done by Fakih et al. (15), as they didn't observe any complication in trans-rectal oocyte retrieval.

In our study, a significant increase in AMH level was observed in the TVOR group compared to the TROR group. The age in the TVOR group is higher than that in the TROR group, but this was insignificant. Other research also reported that due to individual heterogeneity, a certain number of women have discrepancies between age and AMH levels (28, 29). The relatively wide distribution of AMH values in the TROR group [median (IQR) = 0.77 (0.177–3.200)] suggests substantial inter-individual variability in ovarian reserve within this study. Such variability is to be expected in assisted reproductive populations. This heterogeneity may have contributed to variability in AMH levels across groups, with higher values contributing to the upper quartile range. The increase in AMH level in the TVOR group in our study may explain the increased number of follicles retrieved and the IQR of the number of oocytes retrieved in the TVOR group compared to the TROR group. Previous studies have found a strong positive correlation between serum AMH levels and the number of oocytes retrieved (30–33). Seifer et al. (34) found that the patients with the number of oocytes retrieved ≥ 11 had a higher AMH levels than those with number of oocytes retrieved ≤ 6.

Our results showed that the number of follicles and oocytes retrieved were negatively correlated to the age of the subjects in both groups (35, 36). The quantity is determined by the number of mature oocytes that can be stimulated, retrieved, and cryopreserved in a single cycle. Although ovarian reserve cannot reliably predict the likelihood of conception, the present study did not directly assess oocyte quality. Nevertheless, accumulating evidence indicates an association between reduced oocyte competence and an increased incidence of chromosomal abnormalities with advancing maternal age (37, 38). Therefore, the observed reduction in oocyte quantity with age in our study may occur alongside a decline in oocyte quality, as reported in the prior research. Therefore, younger patients who cryopreserve oocytes are more likely to produce chromosomally normal embryos, thereby increasing the likelihood of live delivery after a frozen embryo transfer.

Our results showed a negative correlation between the number of follicles and oocytes retrieved and the BMI of the subjects in both groups (39, 40). Obesity may adversely affect oocytes and the endometrium (41), since obese women have a diminished response to ovarian stimulation, necessitating increased dosages of gonadotropin hormone injections. Consequently, standard medication dosages for obese women may provide an insufficient number of recovered mature oocytes (42).

There is a positive correlation between peak E2 on the triggering day and the number of follicles and oocytes retrieved in both groups (43–48). This relationship is elucidated by the observation that E2 was mostly synthesized by the granulosa cells of the ovarian follicles (49). The potential to utilize serum E2 as a predictive indicator for the anticipated number of follicles during follicle puncture has been validated, suggesting that E2 can be employed in clinical practice to forecast the expected follicle count and determine the optimal timing for ovulation induction (44). Kapoor et al. (50) noted that the meiotic potential of oocytes increases with elevated peak E2 levels, hence significantly enhancing oocyte maturation. This was supported by multiple regression analysis, which showed that markers of ovarian response, especially peak E2 levels, were strongly associated with follicular and oocyte yield, confirming their utility as direct measures of response to stimulation. AMH was not independently associated with follicular yield after adjustment but was strongly associated with oocyte yield in TROR, which could be explained by its role as a baseline measure of ovarian reserve and not a direct determinant of follicular recruitment during stimulation. This finding is consistent with previous data that dynamic intra-cycle markers better predict oocyte and follicle yield (51).

The significant association with procedural factors such as anesthesia time may reflect underlying case complexity or the number of follicles accessed during retrieval. In conclusion, the data suggest that intrinsic ovarian reserve (AMH) and the dynamic endocrine response (peak E2) (52) are the main determinants of oocyte yield in the TROR group and that anthropometric and procedural variables may have a minor independent role when biological determinants are accounted for.

The duration of anesthesia was longer in the TVOR group compared with the TROR group; however, no significant difference was observed in procedural (surgical) time between the two groups. Given that anesthesia time closely parallels procedural duration, this difference is likely attributable to differences in perioperative care, including anesthesia induction and recovery, rather than the procedural duration itself. Accordingly, no inference of procedural advantage can be made, and TROR should be interpreted as not inferior to TVOR with respect to perioperative anesthesia exposure. Ioscovich et al. (53) indicated that anesthesia/analgesia during oocyte retrieval is necessary but should be minimized to avoid adversely affecting oocyte and embryo quality.

In addition, the relationship between anesthesia duration and follicle or oocyte yield observed in regression analysis should be interpreted cautiously, as it may reflect procedural factors rather than a direct biological effect. An optimal analgesic protocol for oocyte extraction should exhibit no detrimental effects on the oocytes, provide quick start and recovery, and facilitate simplicity of administration and monitoring (54). It should be emphasized that no measure of patient discomfort or pain was directly evaluated in the present research. Therefore, any differences related to anesthesia time cannot be attributed to variations in pain perception between the two groups.

The available literature on TROR remains limited. To date, evidence is restricted to a small number of experimental and clinical reports, including an animal study (14), a single human study (15), and a case series (16). In this context, the present study adds to the emerging body of evidence by providing additional clinical data comparing TROR with TVOR.

### 4.1 Limitations and future perspectives

This study has several limitations. First, detailed patient-level factors, including comorbidities, reproductive background factors (e.g., smoking, endometriosis, polycystic ovary syndrome (PCOS), autoimmune conditions, and malignancies), and fertility status, were not fully assessed and may act as potential confounders. Future studies should incorporate PCOS status and broader clinical variables to improve adjustment and interpretability. Second, oocyte quality and patient-reported outcomes, including pain, were not assessed. Third, the relatively small sample size restricts the assessment of rare complications. Finally, microbiological assessment of culture media was not carried out after oocyte retrieval, and therefore the possibility of bacterial contamination, including *Escherichia coli*, was not addressed in TROR. Larger multicenter studies are needed to confirm these findings and further evaluate safety and efficacy.

### 4.2 Strengths of the study

This is the first study comparing the efficacy of the TROR vs. TVOR oocyte retrieval methods. The quantitative results of this study indicate that the transrectal approach facilitates favorable ovarian responses and produces outcomes that are generally consistent with those of traditional transvaginal oocyte retrieval. Furthermore, the strong correlations observed between key cycle biomarkers and the number of follicles and oocytes retrieved reinforce the robustness and internal validity of the data. Importantly, the identified predictors (age, BMI, AMH, and peak E2) have clear clinical relevance for patient counseling, cycle selection, and individualized treatment planning, thereby enhancing the translational value of the findings for clinical decision-making.

## 5 Conclusion

Although transvaginal oocyte collection is the standard method for oocyte retrieval and is confirmed to be the safest and easiest way, for patients with an intact hymen particularly in regions with cultural and religious considerations, trans-rectal oocyte collection is considered another safe and simple route. Given the limited available evidence on TROR, the present results add to the growing but still emerging literature on this technique.

## Data Availability

The original contributions presented in the study are included in the article/supplementary material, further inquiries can be directed to the corresponding authors.
